# Self-care education program improves quality of life in patients with chronic heart failure: A randomized controlled study protocol

**DOI:** 10.1097/MD.0000000000023621

**Published:** 2020-12-11

**Authors:** Jing Wang, Weiwei Li

**Affiliations:** Department of Critical Care Medicine, Shengjing Hospital of China Medical University, Liaoning, China.

**Keywords:** chronic heart failure, protocol, quality of life, self-care

## Abstract

**Objective:**

The objective of our research is to explore the efficiency of self-care education on the life quality in chronic heart failure (CHF) patients.

**Methods:**

The experiment will be implemented from July 2021 to July 2022 and was granted through the Research Ethics Committee of Shengjing Hospital of China Medical University (423507-037). Eighty patients are included in the study. The recruitment criteria of patients includes: the patients have been diagnosed with CHF by physician on the basis of echocardiography; being stabilized in the acute disease state; in accordance with medical record, the patients have no sensory-cognitive problems. Any reason for not participating in education course (such as not wishing to continue taking part in our experiment or discharge from hospital) is regarded as the exclusion criterion. The primary outcome is the patients’ life quality, which is evaluated with Iranian heart failure quality of life questionnaire. Other outcomes include the incidence of hospitalization and total medical cost.

**Results:**

Table 1 suggests the comparison of patients’ life quality between control group and study group after receiving the education of self-care.

**Conclusion:**

The program of self-care education can be regarded as the proper method to improve the life quality in CHF patients.

**Trial registration:**

The protocol was registered in Research Registry (researchregistry6225).

## 1 Introduction

Chronic heart failure (CHF) refers to the abnormal structure or function of the heart, which leads to the reduction of oxygen delivery in the whole body.^[1,2]^ It is a global epidemic disease affecting 26 million people worldwide, with about 800,000 novel cases diagnosed each year.^[3,4]^ The incidence rate is expected to continue to increase as a result of changes in demographic and increasing prevalence of risk factors, for instance, ischemic heart disease, diabetes and hypertension.^[5,6]^ The incidence rate and mortality rate of CHF is equal to that of many cancers, and the life quality is worse than that of most other forms of chronic diseases. The poor outcomes, high cost and high incidence rate make it a significant problem of public health.

The increase in incidence rate, coupled with the increased survival rate caused by advances in health care and medicine, has resulted in an increasing cost of health care systems. In the developed countries, up to 2% of the health-care budget is devoted to chronic heart failure, with a large part of the expenditure is owing to non-compliance with treatment recommendations and hospitalization.^[7]^ In view of the complexity of CHF, patients should have a good understanding of how to manage the symptoms of CHF. Hence, it is necessary to develop the self-care skills of patients, in order to decrease the probability of hospitalization and improve the life quality.^[8,9]^ Self-care is defined as the decisions and actions made by patients to maintain their life, wellbeing and health functioning.^[10]^ This contains taking medications as needed, sticking to diet therapy and exercise, taking proper action and then monitoring symptoms, and keeping daily contact with the health professionals.^[11]^ While this may seem like a simple linear approach on the surface, there is a growing awareness of the complexity of the process. However, few studies have focused on the relevant research and we conduct this randomized controlled study protocol to evaluate the efficiency of self-care education on the life quality in CHF patients.

## 2 Methods

The experiment will be implemented from July 2021 to July 2022 at Shengjing Hospital of China Medical University. The experiment was granted through the Research Ethics Committee of Shengjing Hospital of China Medical University (423507-037) and recorded in research registry (researchregistry6225). Before the registration, the patients who are recruited receive written informed consent.

### 2.1 Inclusion and exclusion criteria

Eighty patients are included in the study. The recruitment criteria of patients includes: the patients have been diagnosed with CHF by physician on the basis of echocardiography; being stabilized in the acute disease state; in accordance with medical record, the patients have no sensory-cognitive problems. Any reason for not participating in education course (such as not wishing to continue taking part in our experiment or discharge from hospital) is regarded as the exclusion criterion. Sequence of random numbers is generated by a computer. Sequentially numbered sealed opaque envelopes are used for the concealment of random numbers. All the patients participating in this study are randomly divided into control group and study group, with 40 patients in each group.

### 2.2 Intervention

In control group, the patients are given conventional education by the experienced nurses and offered the educational pamphlets when discharged from hospital. Nevertheless, in addition to routine education, in intervention group, patients are given program of self-care education, including a total of 3 sessions, lasting for 2 months. The program of self-care education is conducted through the first researcher, a well-experienced nurse, for 2 consecutive days. The time of each session varies from 40 minutes to 50 minutes, depending on the needs of the participants. Moreover, each class is conducted separately and is taught utilizing the PowerPoint slides. In addition, the educational manual is distributed to patients. The program of self-care education is composed of information on cardiac physiology and anatomy, risk factors for CHF, diagnosis and symptoms, disease management, exercise and diet, medications along with their relevant side effects, as well as the monitor of blood pressure. The content of this education is compiled via the literature review, and the effectiveness of the content is confirmed through the expert panel. In intervention group, the patients are followed up by telephone every month after discharge to ensure that the content is being conducted. After collecting the data, the content of the self-care education program is offered to the participants in the control group.

### 2.3 Outcome measures

The primary result is the patients’ life quality, which is evaluated with Iranian heart failure quality of life questionnaire. Other outcomes include the incidence of hospitalization and total medical cost.

### 2.4 Statistical analysis

The analysis of all the data are conducted with the software of IBM SPSS Statistics for Windows, version 20 (IBM Corp, Armonk, NY). Afterwards, all the data acquired are represented through the appropriate characteristics, for example, standard deviation, mean, median as well as percentage. And independent *t* tests and χ^2^-tests are respectively utilized to analyze the categorical variable and continuous variable. *P* value less than .05 indicates that there is statistical significance.

## 3 Result

Table 1 suggests the comparison of patients’ life quality between control group and study group after receiving the education of self-care.

**Table 1:** The comparison of patients’ life quality between control group and study group after receiving the education of self-care.

Study group (n = 40) Control group (n = 40) *P* value
Symptoms and their severity
Physical limitations
Social interference
Psychological condition
Self-efficacy and knowledge
Life satisfaction
Incidence of hospitalization
Total medical cost

## 4 Discussion

The objective of our research is to explore the efficiency of self-care education on the life quality in CHF patients. CHF is a debilitating and progressive disease, and its prevalence is increasing, affecting both developing countries and developed countries.^[12,13]^ It is related to increased hospitalization frequency, shortened life expectancy, as well as poor life quality, and is the most prevalent heterogeneous clinical syndrome and burden in the elderly. The self-care education and modification of lifestyle are the significant strategies for all the patients.^[14,15]^ Published research have defined the self-care as a process of natural decision-making that includes response to symptoms (management) when symptoms appear and the selection of behaviors to keep the physiological stability (maintenance).^[16]^ The programme of self-care education can in-depth develop the personal abilities and encourage her or him to adhere to a treatment regime.^[17]^ The program also can help the CHF patients control their symptoms, shorten hospital stays, enhance their self-confidence, and then improve their life quality. In fact, the program usually improves the patient's compliance with the process of treatment, thus improving the quality of treatment.^[18]^ Nevertheless, more research is needed to develop proper approaches to improve the life satisfaction and physical limitations of CHF patients.

## 5 Conclusion

The program of self-care education can be regarded as the proper method to improve the life quality in CHF patients.

## Author contributions

**Conceptualization:** Weiwei Li.

**Data curation:** Weiwei Li.

**Funding acquisition:** Weiwei Li.

**Writing – original draft:** Jing Wang.
